# *In vivo* murine breast cancer targeting by magnetic iron nanoparticles involving L. GG cytoplasmic fraction

**DOI:** 10.22038/ijbms.2021.54961.12322

**Published:** 2021-05

**Authors:** Salar Mokriani, Amir Tukmechi, Naser Harzandi, Leila Jabalameli

**Affiliations:** 1 Department of Microbiology, Faculty of Sciences, Karaj Branch, Islamic Azad University, Karaj, Alborz Province, Iran; 2 Department of Microbiology, Faculty of Veterinary Medicine, Urmia University, Urmia, West Azarbaijan Province, Iran; 3 Department of Microbiology, Faculty of Veterinary Medicine, Karaj Branch, Islamic Azad University, Karaj, Alborz province, Iran

**Keywords:** Anti-cancer, Drug delivery, In vivo, Nanoparticles, Probiotic, 4T1

## Abstract

**Objective(s):**

Use of chemical anti-cancer drugs frequently creates serious side effects. However, probiotics are natural and treat different kinds of cancer without undesired effects.

**Materials and Methods:**

In this study, a nano delivery system was planned to transport the *Lactobacillus rhamnosus* GG (L. GG) cytoplasmic fraction (Cf) to cancerous tissue in a mouse model. Magnetic iron nanoparticles (MINPs) were synthesized and loaded with L. GG-Cf(0, 0.312, 0.625, 1.25, and 2.5 mg/ml) and were administrated for three weeks to treat experimentally induced murine breast cancer in a constant magnetic field. At the end of the trial, the treating efficacy of this complex molecule was evaluated via western blotting, immunohistochemistry, and qPCR.

**Results:**

Results showed that MINPS can deliver and accumulate L. GG-Cf in cancer tissue, and reduce the size and volume of the tumors. Additionally, in cancer tissues of treated mice with 2.5 mg/ml of Cf-MINPs, significantly induced apoptosis was seen compared with untreated mice (control), and our data proved that this induction may be due to the caspase-3 pathway.

**Conclusion:**

L. GG-Cf could treat murine breast cancer, and MINPs are a suitable candidate for drug delivery because of their safety, uniformity, and magnetic properties.

## Introduction

Breast cancer is the most common type of malignancy and is still the second main cause of cancer deaths among women (1). Despite the recent advances in treatment, metastasis remains the main cause of mortality, and breast cancer trends to invade the other organs. Unfortunately, metastatic tumors are usually inaccessible by surgery or radiotherapy, and there are currently no effective therapies to treat breast cancer metastasis. Therefore, it is essential to develop new therapeutic agents to prevent and treat metastatic breast cancer (2, 3).

Probiotics are defined as ‘dietary supplements, containing viable non-pathogenic microorganisms or their derivatives which give health benefits to the host’. Currently, the anti-cancer properties of probiotics are demonstrated and their active role in treatment is clear. Several strains belonging to *Lactobacilli *are effective in the suppression of cancer incidence (4, 5). Goldin and Gorbach were among the first to demonstrate the association between a diet enriched with *Lactobacillus rhamnosus* GG(L.GG) and reduced incidence of colon cancer. *In vitro* studies on L. GG as both viable and heat-inactivated bacteria as well as homogenate and cytoplasm extracts, demonstrated significantly decreased cell proliferation, induced apoptosis, and influenced the polyamine metabolism of some human gastrointestinal (GI) cancer cells (4, 6, 7).

Recent advances in nanotechnology are now driving a revolution in cancer treatment, and iron nanoparticles were some of the first nanomaterials for use in oncology. Magnetic nanoparticles (MNPs) are a kind of smart magnetic nano-magnetic material with small particle size and large specific surface area. The most frequently used nanomaterial is the iron oxide nanoparticle, including Fe_3_O_4_ and Fe_2_O_3_. It is well known that MNPs have an important role in cancer drug delivery and treatment (8). Due to the small size and large specific surface area of MNPs, they can easily reach the site of cancer. Therefore, application of MNPs for drug delivery cannot be ignored. By *in vivo* application of an external magnetic field, we could concentrate the anti-cancer drugs at the target organs. Hence, MNPs are used as drug carriers by binding to the chemotherapeutic agents. Commonly, chemotherapeutic drugs are loaded in MNPs, and used in cancer treatment (9). 

In general, most chemotherapeutic agents can damage healthy tissues and cells. Typical toxic side effects include myelosuppression, mucositis, stomatitis, alopecia, and some severe complications, such as cardiotoxicity and hepatotoxicity (10). To overcome this problem, there have been several approaches already proposed with the aim of reducing systemic toxicity. One strategy is the use of speciﬁc nanotherapeutic delivery systems, and the other promising approach consists of the use of magnetic iron oxide nanoparticles (MNPs) in their double function as a carrier and inductor of magnetic heating (magnetic hyperthermia). MNPs usually consist of a core of biocompatible iron oxides and are coated with a shell of polysaccharides or other biocompatible molecules. When exposing them to an alternating magnetic ﬁeld (AMF) of appropriate amplitude and frequency, they release heat due to loss processes during the reorientation of the magnetization in the magnetic ﬁeld, or due to frictional losses in the case of the nanoparticle being able to rotate in the surrounding medium (11). 

Based on these findings, the idea has been put forth that loading magnetic iron nanoparticles (MINPs) with a cytoplasmic fraction of L. GG might represent a potentially powerful novel approach to the treatment of breast cancers. We believe the use of combined therapeutic platforms has provided favorable consequences for the treatment of breast cancer. To this end, it has been attempted to employ MINPs as carriers for L. GG cytoplasmic fraction delivery and to induce hyperthermia at the location of the lesion by an external magnetic field, for the treatment of breast cancer (12). 

## Materials and Methods

Preparation of* L. rhamnosus* GG cytoplasmic fraction 

L. GG strain (ATCC 53103) was bought from the micro-organisms collection of the Scientific and Industrial Research Organization of Iran. To prepare the cytoplasmic fraction, the bacterium was cultured in 100 ml of MRS broth and incubated at 30 °C for 18–24 hr under a 5% atmosphere of CO_2_. After the incubation period, the bacterium was centrifuged for 15 min at 3500 rpm and 4 °C, and the collected sediment was washed twice by sterile normal saline. Then a suspension of the bacterium was prepared in PBS (phosphate buffer solution, 0.1 M and pH 6.9), and cell concentration was adjusted at 10^9^ CFU/ml by MacFarland standard tube. The bacterial suspension was crushed using a sonicator device (Soniprep 150, UK) beside the ice at 10 rounds, 1 min/round, 70% amplitude, 50W. Finally, the samples were centrifuged for 30 min at 10000 rpm round at 4 °C. The supernatant was removed and the protein concentration of *L*. GG cytoplasmic fraction was determined using a protein assay kit (Pars Azmun, Iran) according to the manufacturer’s instructions. The concentration for cytoplasmic fraction was approximately 2.50 mg protein/ml (13).

***Synthesis of MINPs and Cf-MINPs***

MINPs were synthesized based on the method described previously*.* FeCl_3_.6H_2_O (0.54 g), sodium acetate (1.5 g), and sodium acrylate (1.5 g) were dissolved in 20 ml of ethylene glycol (14). After vigorous stirring of the mixture for 1 hr, the homogeneous solution was transferred to a Teflon-lined stainless-steel autoclave (25 ml volume), sealed, and heated to 200 °C. After a 10 hr reaction period, the autoclave was cooled to room temperature. The obtained Fe_3_O_4_ spheres were washed with water and ethanol and dried in a vacuum for 15 hr. Then, the MINPs were covered by an SiO_2_ layer based on the method of Sharifi *et al*. Briefly, 5 ml of the aqueous solution of MINPs was diluted with 60 ml of ethanol and one ml of water. The blend was homogenized by ultrasonication for 40 min before adding 2 ml of ammonia solution. After 40 min, 0.2 ml of tetraethyl orthosilicate solution dissolved in 10 ml of ethanol was injected, and the reaction was carried out for 120 min and the final product was gathered with a magnet. Then, nanospheres were washed several times with ethanol and water, followed by drying in a vacuum for 15 hr. Afterward, the MINPs coated with SiO_2_ were heated in an oven with a heating rate of 5 °C/min to reach 700 °C for 5 hr. After cooling at room temperature, the product was dissolved in 0.5 M NaOH for 7 hr. Finally, the MINPs were gathered by magnetic methods and washed three times with water and ethanol (15).

***Cf loading and assessment of releasing***

For Cf loading, the Fe_3_O_4 _nanospheres (40 mg) were added into 10 m1 of Cf solution (2.5 mg/ml) at room temperature. The mixture was stirred for 24 hr, and then the Cf-loaded MINPs were magnetically separated (14). At first, a suspension of the Cf-MINPs was prepared in PBS then Cf releasing was done for 720 min incubation at 37 °C and various pH (3, 5, and 7; the pH was adjusted by sodium hydroxide). So, 1 mg Cf-MINPs was dissolved in 10 ml of PBS and every 60 min 2.5 ml of solution was extracted for measurement by absorbance at 480 nm until 24 hr. The release of Cf was estimated based on the following equation (15):

Cf release %={((5×)+(50×C_n_))/ amount of Cf in MINPs}×100

Where, C_i_ and C_n_ refer to the Cf concentration at time of initiation and 6 hr, respectively.

The encapsulation efficiency (%) was then determined by the following formula:

encapsulation efficiency (%)= [(A-B)/B]×100

Where A is the total amount of L. GG cytoplasmic fraction and B is the amount of CF remaining in the solution.

***Characterization***

In this study, the morphology of Cf-MINPs was characterized using a transmission electron microscope (TEM) and a scanning electron microscope (SEM). For the TEM photographs, a Philips microscope model CM 100 was used at an accelerating voltage of 120 kV. Also, the SEM image was recorded in a JSM-7900F scanning electron microscope flagship model with a field emission gun (FEG). Notably, Cf-MINPs anti-cancer activity was evaluated against the 4T1 cell line *in vitro* (the data are in press).

***Animal Model and Cf-MINPs administration***

Seven-week-old, female BALB/c mice were purchased from the Animal Center of Pasteur Institute of Iran and maintained under speciﬁc pathogen-free conditions. In this study, the experiment and animal care procedures were approved by the Animal Ethics Committee of Faculty of Veterinary Medicine, Urmia University, Urmia, Iran (Ref No. AECVU-102-2019) and were performed under the National Research Council Guide for Care and Use of Laboratory Animals (16). The 4T1-based breast cancer model was applied as reported by Lu *et al*. (17). Briefly, 100,000 4T1 cells were suspended in 100 µl RPMI and the cell suspensions were injected subcutaneously close to the nipples of the mice. When breast tumors became palpable, animals received Cf-MINPs treatments via peritumoral injections (30 μl) for three weeks. Doses were the following: 0.312, 0.625, 1.25, and 2.5 mg/kg Cf loaded into MINPs. A magnetic field of 4 kA/m amplitude (2 kW, 540 kHz) was used for 4 min. All mice every three days were anesthetized and placed under the magnetic field for 4 min after 8 hr of injection (15). During the experiment, tumor volume was quantified using a caliper (Stahlwille, Germany) and the following formula:

Tumor volume (cm^3^)=(W2×L)/2

Where W is tumor width, L is tumor length.

Capecitabine (Roche, Germany) was applied peritoneally (6 mg/kg dose), and sterile normal saline was used as control. After 3 weeks of treatment (day 28 post-inoculation), four mice were randomly selected from each group and sacriﬁced. The breast tumor was collected and ﬁxed in 10% neutral buﬀered formalin. During the treatment, the tumor size of each mouse was measured every other day.

***Measurement of ROS in mice breast tumor***

Detection of ROS was performed by a Fluorometric Assay Kit (MyBioSource, USA). Small pieces of fresh breast tumors were embedded into optimal cutting temperature (OCT) solution (Fisher Scientific, USA), and cut into 5 µm-thick sections by a semi-automatic microtome (Radical, India). The levels of ROS were determined in accordance with the protocol provided by the manufacturer, and fluorescence was read with a fluorescence plate reader (Promega, USA) at excitation 500  nm and emission 525 nm (15).

***Immunohistochemistry***

Caspase-3 protein was detected in mice breast tumors using a biotin-streptavidin-immunoperoxidase method (18). Frozen sections 5 μm thick were blocked with normal goat serum (Vector, USA), 1:10 dilution for 15 min, and incubated with rabbit anti-caspase-3 polyclonal antibody (MyBioSource, USA), 300 µl/ml, 1:50 dilution for 30 min. Biotinylated goat anti-rabbit IgG antibody, 1500 dilution, and peroxidase-conjugated streptavidin (Sigma, USA) were applied to the tissue for 10 min, respectively. 3,3-diaminobenzidine was used as the substrate chromogen. Then sections were stained with hematoxylin, and part of untreated healthy mice mammary gland was used as the negative control. The positive cells in immunochemistry staining were detected by brown-colored cytoplasm under a light microscope and counted with a reticular lense (19-21).

***Western blot analysis***

10 grams of fresh breast tumor tissue were lysed with a homogenizer in RIPA lysis (radioimmunoprecipitation assay buffer; Abcam, UK) buffer containing phenylmethanesulfonyl ﬂuoride (PMSF; Goldbio, USA) beside the ice. The lysates were centrifuged at 12,000 rpm for 15 min at 4 °C and denatured by boiling in a loading buﬀer (Sigma, USA). After protein assay of the lysate, an equal amount of 30 µg protein was loaded and separated on 12% glycine SDS-PAGE gel, and transferred to polyvinylidene diﬂuoride membranes (0.45 µm; Sigma, USA). The membranes were then incubated with monoclonal antibody against cleaved caspase-3 antibody (ab214430, 17 kDa) (Abcam, Cambridge, UK) for 1 hr. For loading control, the membranes were probed with anti-β-actin (ab8227, 42 kDa) (Abcam, Cambridge, UK) antibody for 1 hr. The membranes were then incubated with Horseradish peroxidase (HRP)-conjugated anti-rabbit secondary antibody (Abcam, UK) for 1 hr. The luminol reagent and peroxide solution were used as the substrate for HPR and the immune-complex on the membrane was detected using a chemiluminescence (CL) image analysis apparatus (Bio-Rad, USA) (22).

***Expression of caspase-3 gene***

Total RNA from the tumor samples was obtained by using the TRIzol reagent (YektaTajhiz, Iran), and the quality and quantity of extracted RNA were analyzed using a NanoDrop (Bio-Rad USA). The complementary DNA (cDNA) was synthesized from 2 µg total RNA by SinaClon First Strand cDNA synthesis Kit (Iran) according to the manufacturer’s guideline.

Quantitative real-time PCR (qPCR) was done with an Applied Biosystems 48 well StepOne™ (Thermo Fisher Scientific, USA), using a final reaction volume of 20 µl containing 0.3 µmol/l of each forward and reverse primer (Table 1), 1x SYBR Green master mix (YektaTajhiz, Iran), and 0.2 µl of cDNA. β-actin was used as internal control housekeeping gene expression. The reaction was performed as 1 cycle at 95 °C for 10 min, 40 cycles at 95 °C for 10 sec, 64 °C for 30 sec, and 72 °C for 15 sec (23, 24). 

Quantitative analysis was done by measurement of the threshold cycle (CT) values during the exponential phase of amplification. ΔCT was calculated by the difference between the CT values of caspase-3 and the β-actin gene. Relative quantifications were calculated as 2- ΔCT/(average of (2- ΔCT)) (24).

***Statistical analysis***

The results are expressed as mean ± Standard Error of the mean (SEM). One-way analysis of variance (ANOVA) test followed by Tukey’s test was used for comparing the means. Independent student’s t-test was used to compare statistical differences between the means of caspase-3 and beta-actin gene expressions in Cf-MINPs treated and untreated mice. All statistical analyses were performed using version 22 of SPSS, and *P*≤0.05 was considered to be signiﬁcant difference.

## Results

***Capecitabin and Cf release***

**Figure 1:**
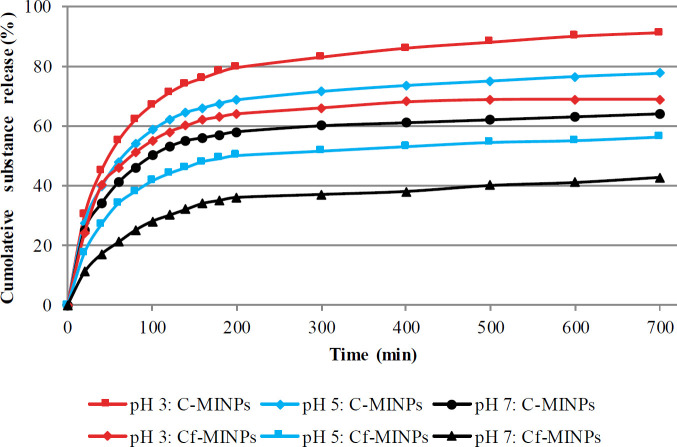
Assessment of Cf and capecitabin release at 37 °C at various pH

The results showed that capecitabin release from Cap- MINPs follows a pH-dependent release form. Furthermore, about 91.2±3.81% of capecitabin was released from Cap-MINPs at pH 3, while 77.6±3.48% and 64±4.01% of the substance were released at pH 5 and 7 after 720 min, respectively (Figure 1). Also, Cf was released from Cf-MINPs as 56.2±3.17%, 58.1±2.98%, and 42.7±2.52% in pH 3, 5, and 7, respectively. Chiefly, substance release at pH 3 was much faster and higher than pH 7. As shown in Figure 1, the maximum release was 46.1±3.08% in acidic pH and 21.4±1.77% in the neutral pH at 60 min. Obtained results greatly suggest pH-sensitive substance release in the instance of Cap-MINPs and Cf-MINPs.

***Nanoparticle characterization***

**Figure 2:**
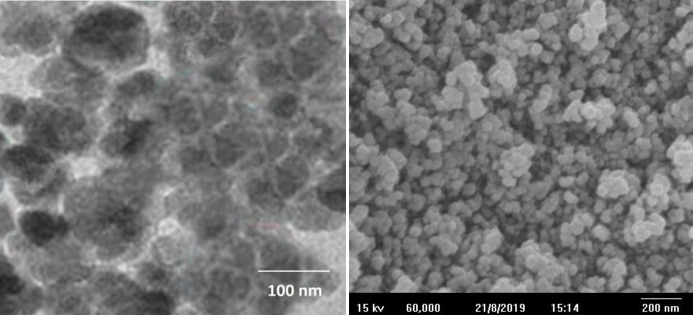
Morphology and size of MINPs: A) TEM and B) SEM

The morphology and size of the synthesized MINPs were analyzed by TEM (Figure 2A) and SEM (Figure 2B), respectively. The obtained results using SEM imaging clearly show that Cf-MINPs have a spherical shape, and the average particle size is around 109 nm. TEM images show a very constant size distribution of MINPs.

***Tumor volume measurement***

**Figure 3:**
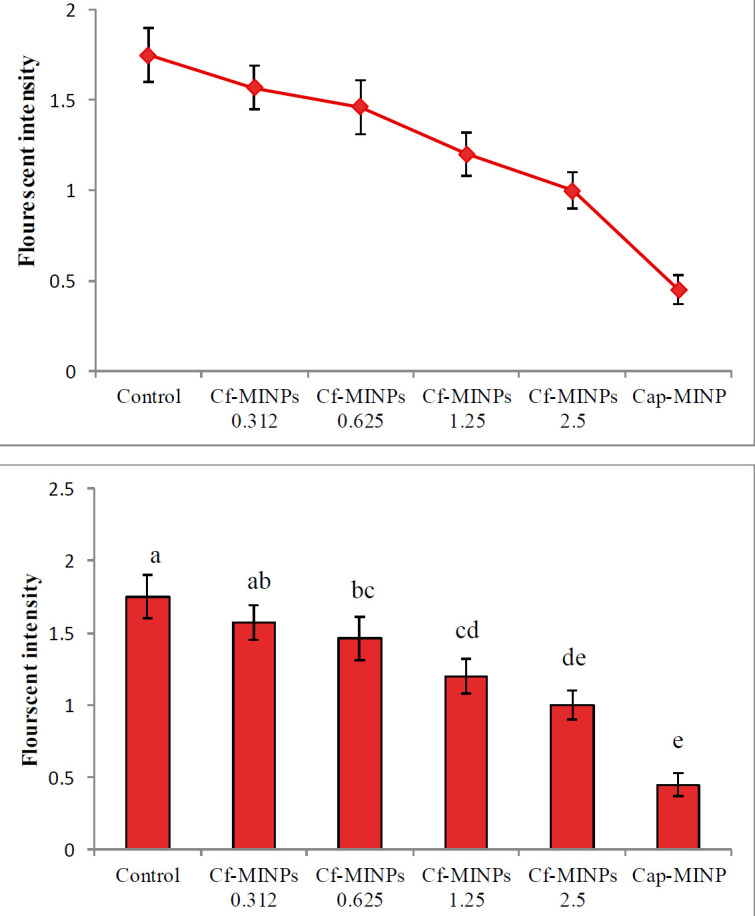
Measurement of reactive oxygen species (ROS) production from control and treated mice (A, B). Significant differences among groups are shown by different letters (*P*<0.001)

Tumor growth from weeks 1–4 was significantly slower in the Cap-MINP mice treated with 2.5 mg/ml of Cf-MINPs compared with other groups (Figure 3). At the end of the experiment (day 28), the maximum and minimum tumor volumes were related to the control (1.02±0.01 cm^3^), Cap-MINP (0.08±0.02 cm^3^), and Cf-MINPs (0.11±0.03 cm^3^), respectively. 

***ROS measurement***

**Figure 4:**
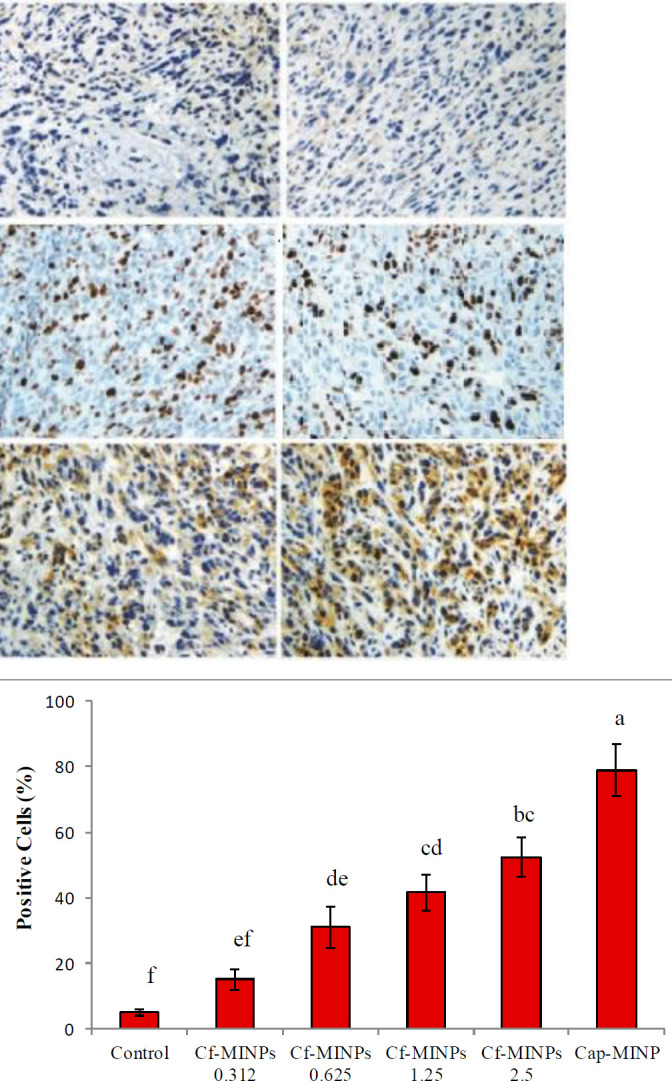
Immunohistochemistry (IHC) staining for caspase-3 of murine breast tumors. A: Untreated group, B-E: 0.312, .625, 1.25, and 2.5 mg/ml of Cf-MINPs and F: Cap-MINPs. Significant differences among groups are shown by different letters (*P*<0.001)

After three weeks of treatment with Cf-MINPs and Cap-MINPs the levels of reactive oxygen species were evaluated in mice breast cancer specimens by a fluorometric assay kit. Interestingly, all concentrations of Cf-MINPs could reduce the level of ROS in tumor tissue (Figure 4). It could be noticed that the ratio of ROS reduction in treated mice with Cf-MINPs follows a concentration-dependent profile. Treatment with 2.5 mg/ml of Cf-MINPs significantly reduced the quantity of ROS, compared with other treatment groups, in breast cancer tissue. 

***Immunohistochemistry***

**Figure 5:**
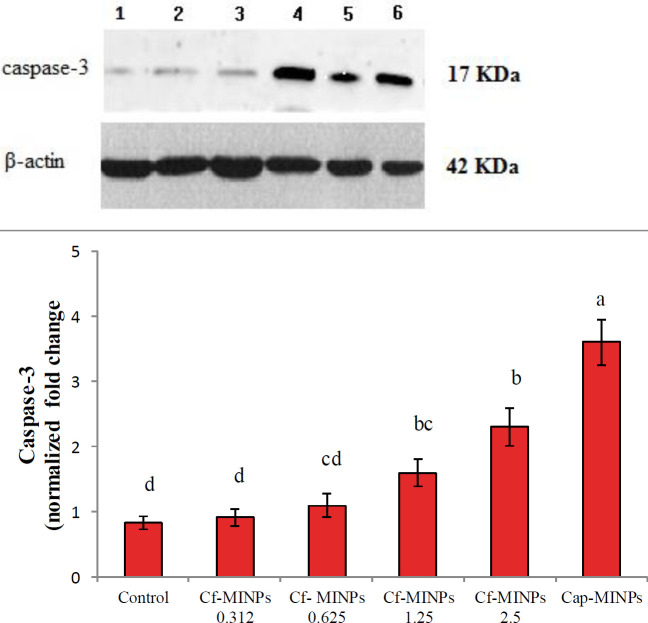
Significant differences among groups are shown by different letters (*P*<0.001)

Results showed that administration of different amounts of Cf-MINPs significantly result in overexpression of caspase-3 protein in breast cancer tissue. The specific antibody for caspases-3 (Figure 5) selectively stained the cytoplasm of breast cancer tissue, and nuclear morphology was consistent with apoptosis. It is clear that this antibody recognized activated protein at the primary stage of apoptosis. Counting the cells with brown colored cytoplasm showed administration of 2.5 mg/ml Cf-MINPs significantly induces apoptosis in cancer tissue. Breast cancer samples of the mice treated with 2.5 mg/ml of Cf-MINPs had a higher number of apoptotic cells than the control. Also, capecitabine acts as Cf-MINPs to induce apoptosis in the cancerous tissue.

***Western blot analysis of caspase-3 of breast cancer***

**Figure 6:**
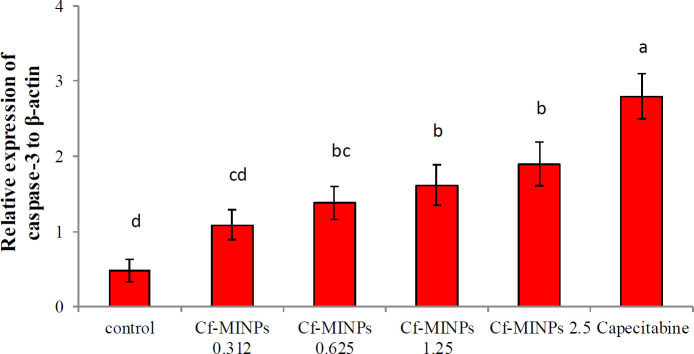
Effect of different concentrations of Cf-MINPs and Cap-MINPs on expression of Caspase-3 in mice breast cancer tissues. Significant diffe rences among groups are shown by different letters (*P*<0.001)

Western blot analysis demonstrated that in tumors sampled from different amounts of Cf-MINPs treated mice a higher level of caspase-3 was detected compared with breast cancer from untreated mice (Figure 6). The levels of caspase-3 were significantly higher in tumor tissues of mice treated with 2.5 mg/ml Cf-MINPs compared with the other groups. Consequently, the levels of caspase-3 in cancer tissues of 2.5 mg/ml Cf-MINPs treated mice were 2.3 folds higher compared with the untreated.

Cf-MINPs increased the Caspase-3 expression gene in tumor tissues. qPCR was conducted to explain the mechanism of cell death induced by Cf-MINPs. After three weeks of treatment, breast cancer tissue was collected from all mice, and expression of the caspase-3 gene related to apoptotic pathways was analyzed. There was a significant increase in Caspase-3 expression in Cf-MINPs and capecitabin groups (Figure 7). The qPCR analysis revealed that increased level of caspase-3 expression follows a dose-dependent profile. Besides, the caspase-3 expression level was significantly higher than in the other groups. Also, we did not notice any differences between the level of caspase-3 expression in mice treated with 2.5 mg/ml of Cf-MINPs and capecitabin at *P*≤0.05 significance level. 

## Discussion

Our data showed that loading *L. rhamnosus* GG cytoplasmic fraction on MINPs significantly inhibited breast cancer enlargement by inducing apoptosis. More studies proved that administration of lactic acid bacteria (LAB) caused inhibition of proliferation and induction of apoptosis in the tumor. Researchers showed that cytoplasm fractions of LAB have potential antiproliferative effects against some cancer cell lines. These scientists explained that the genuine components have antiproliferative activity in cytoplasm fraction, and their action mechanism has been unknown till now (25). A study identified an 8 kDa protein isolated from *L. rhamnosus* (LR), that could suppress the growth of colorectal cancer. They speculated that p8 suppresses tumor growth by inducing cell cycle arrest (26). Hassan proposed that the metabolites, from *Lactobacilli*, contain a number of peptides, amino acids, short-chain fatty acids, lactic acid, butyric acid, and certain other chemicals, which may play key roles in its anti-cancer activities (27).

Currently, chemotherapy is the standard cancer treatment; although, it may not be the ideal therapy as it is associated with complicated side effects on patients (28). The use of probiotics to treat cancer may decrease the risk and severity of chemotherapy-related toxicity and side effects associated with treatment. Hence, we used the cytoplasmic fraction of L.GG to treat breast cancer in mice because of its safety and anti-cancer activity. The major limitation for cytoplasmic extraction is that a small amount of cytoplasmic fraction will be obtained from a high volume of cultured bacteria. As a result, in this manner, administration of cytoplasmic fractions may not be enough for cancer treatment in the body. On the other hand, the detoxification activity of the liver may eliminate a huge part of cytoplasmic fraction from the bloodstream and reduce the Cf shelf life. For solving this problem we decided to transfer the L.GG cytoplasmic fraction to the tumor site by a natural vehicle. Many studies have documented that nanoparticles especially magnetic iron oxide nanoparticles are suitable for this purpose. 

Today advances in nanotechnology are driving a revolution in cancer therapy; iron oxide nanoparticles were the first nanomaterials to see application in oncology. Researchers suggested that activating localized iron oxide nanoparticles via AMF could treat cancer *in vitro* and *in vivo* (29). In addition to killing cancer cells directly, hyperthermia can enhance the efficacy of Cf-MINPs, and our *in vivo* experiments suggested an observable reduction of tumor volumes, particularly after treatment with 2.5 mg/ml of Cf-MINPs. These effects were most detected between days 12 and 19 after treatment.

To accomplish biological demand, the ideal anti-cancer compounds should be biocompatible. MINPs have good water distribution, which indicates the potential biosafety of these particles. In our previous work, the cytotoxicity of MINPs was evaluated on Madin-Darby Canine Kidney (MDCK) cells by MTT assay (in press). Based on our results MINPs containing different amounts of CF could inhibit growth of the 4T1 cell line. The inhibitory effects were started after 24 hr and reached the maximum at 48 hr after treatment, then this property was slowly reduced in 96 hr. The most inhibitory effects were seen in the 4T1 cell line challenged with MINPs containing 1.25 mg/ml of CF after 72 hr. Also, results proved that MINPs without CF and CF alone had inhibitory properties against the 4T1 cell line, and higher inhibition of growth was seen after 72 hr of treatment. Statistical analysis showed MINPs containing 1.25 mg/ml of CF significantly inhibit the growth of 4T1 compared with the cells that were treated with MINPs without CF and CF. Also, we observed that MDCK cell viability was not adversely aﬀected at amounts up to 40 mg/ml for the particles. However, MINPs exhibited lower cell toxicity, which proposed that the nanoparticles are biocompatible and suitable for medical applications.

It was investigated that, after intraperitoneal administration, MINPs will quickly penetrate across the blood vessels and reach into the bloodstream because of their particle sizes (29). Hence, MINPs are immediately distributed in all crucial organs, such as the liver, kidney, and spleen, whereas, by using a magnetic field the nanoparticles accumulate in tumor tissue (mice breast). In this regard, researchers demonstrated that use of magnetic fields not only reduces accumulation of anti-cancer drugs in the major organs but also increases drug performance by increasing drug concentration in the tumor tissue (30). As we showed, the Cf release was quite fast during the ﬁrst 60 min, but decreased with time and pH, and came to a plateau at 63.1% in pH 7 after 720 min. This is in agreement with Benyettou *et al.* and Gholami, *et al. *revealing that the highest rate of drug release from a vehicle happened in an acidic environment (31, 32). Therefore, Cf-MINPs can be useful in magnetic field-based drug delivery, especially cancer therapy.

Also, in this study, the effects of Cf-MINPs on the rate of ROS production were evaluated in mice breast cancer. As shown in Figure 4 all amounts of Cf-MINPs decreased the level of ROS in tumor tissue, a significant decrease was observed in tumor samples especially for 1.25 and 2.5 mg/ml Cf-MINP three weeks after treatment. To date, many studies have investigated the effects of probiotics on ROS production. For example, *L. fermentum*, *L. coryniformis*, and *L. gasseri* can reduce the levels of ROS in tumor tissues via decreasing the NADPH oxidase activity (33). NADPH oxidase (NOX) complex is considered to be the main source for ROS generation (34). Another mechanism for the anti-oxidant activity of probiotics is production of various metabolites like glutathione (GSH), butyrate, and folate (35). Similarly, it has been reported that some Lactobacilli induce increased activity of anti-oxidative enzymes, including superoxide dismutase (SOD), glutathione peroxidase (GPx), glutathione reductase (GR), peroxidase, SGT, and catalase (CAT) (34). They also promote production of certain antioxidant biomolecules, such as EPS, and exhibit metal chelating activity (33).

It is now clear that many probiotics can kill tumor cells by activating ordinary apoptotic pathways. Caspase-3 is a member of the cysteine protease family, which plays a key role in affecting apoptosis (35). Negative cells for expression of caspase-3 had blue-stained nuclei, but positive cells appeared dark brown without any deformity of their nuclei. Our results showed injection of Cf-MINPs can increase the expression of caspase-3 in breast cancer. The higher expression of caspase-3 was significantly detected in mice treated with 2.5 mg/ml Cf-MINPs (Figure 5). Our data are in agreement with Lin *et al*. who showed L. GG can induce expression of caspase-3 in mouse intestinal cells (36), as well as with Sharma *et al*., who proved that cell lysate of *L. acidophilus* induces apoptosis via caspase-3 expression in mice hepatocytes after experimental toxicity with acetaminophen (37). 

Our results show that assessment of active caspase-3 by western blotting matched up with the level measured by immunohistochemistry. These results propose that measurement of the active caspase-3 is required to identify ongoing apoptosis. Figure 6 shows that the breast tumor sampled from Cf-MINPs (2.5 mg/ml) treated mice was detected with a 2.3 fold higher level of active caspase-3 compared with the tumor from untreated mice (control). It is noticeable that caspase-3 is a critical mediator of apoptosis and correlates with apoptotic rates in breast cancer. Indeed, it is a potential marker for predicting response to chemotherapeutic substances in breast cancer, and obtained data showed that L. GG cytoplasmic fraction induces apoptosis in an experimental model of murine breast cancer via the active caspase-3 pathway.

For the first time, we report a cytoplasmic fraction from *L. rhamnosus* GG that can up-regulate transcription of caspase-3 in 4T1 induced murine breast cancer. The qPCR results show treated mice with 2.5 mg/ml of Cf-MINPs had a significantly higher mRNA level of the caspase-3 gene. Although many researchers indicated that heat-killed probiotics (6) and their fraction (cell-free supernatant) (24) can increase mRNA levels of caspase-3 gene in the tumor cell line, we demonstrated that if L. GG cytoplasmic fraction was loaded on the MINPs, it could induce apoptosis *in vivo* via activation of the caspase-3 protein.

**Table 1:** Primer sequences of caspase-3 and Beta-actin for qPCR

Gene	Forward primer (5’-3’)	Reverse primer (5’-3’)
Caspase-3	GTTGTGAGTTCTGGTTTGTGTGG	GATGCTTTTCCAAGTCTGTGTG
Beta-actin	AATTCCATCATGAAGTGTGA	ACTCCTGCTTGCTGATCCAC

MINPs: magnetic iron nanoparticles; TEM: transmission electron microscope; SEM: scanning electron microscope

Cf: Cytoplasmic fraction, MINPs: Magnetic Iron Nanoparticles and Cap: Capecitabine

Cf: Cytoplasmic fraction, MINPs: Magnetic Iron Nanoparticles and Cap: Capecitabine

Cf: Cytoplasmic fraction and MINPs: Magnetic Iron Nanoparticles

## Conclusion

In this study, we used an approach for the synthesis of constant MINPs loaded with Cf. MINPs which were particularly suitable for potential targeted drug delivery to breast tumors in a magnetic field. The results showed that Cf-MINPs can directly accumulate in the cancer tissue. The consequences from *in vivo* experiment showed that Cf-MINPs induced apoptosis, and immunohistochemistry, western blotting, and qPCR results confirmed that 2.5 mg/ml of L. GG Cf could result in significant increase in the level of caspase-3 in breast cancer. At the end of three weeks’ treatment, Cf-MINPs could significantly reduce the volume and size of the breast tumors by inducing apoptosis.

## Funding

No funding was received.

## Compliance with Ethical Standards

Animal experiments were performed according to the National Research Council Guide for Care and Use of Laboratory Animals and were approved by the Animal Ethics Committee of Faculty of Veterinary Medicine, Urmia University, Urmia, Iran (Ref No. AECVU-102-2018).

## Conflicts of Interest

The authors declare that they have no conflicts of interest.
